# Ethanol and Acetic Acid Production from Carbon Monoxide in a *Clostridium* Strain in Batch and Continuous Gas-Fed Bioreactors

**DOI:** 10.3390/ijerph120101029

**Published:** 2015-01-20

**Authors:** Haris Nalakath Abubackar, María C. Veiga, Christian Kennes

**Affiliations:** Chemical Engineering Laboratory, Faculty of Sciences, University of La Coruña, Rúa da Fraga 10, 15008 La Coruña, Spain; E-Mails: harisnalakath@yahoo.com (H.N.A.); veiga@udc.es (M.C.V.)

**Keywords:** acetic acid, bioethanol, carbon monoxide, *Clostridium autoethanogenum*, syngas, waste gas

## Abstract

The effect of different sources of nitrogen as well as their concentrations on the bioconversion of carbon monoxide to metabolic products such as acetic acid and ethanol by *Clostridium autoethanogenum* was studied. In a first set of assays, under batch conditions, either NH_4_Cl_,_ trypticase soy broth or yeast extract (YE) were used as sources of nitrogen. The use of YE was found statistically significant (*p* < 0.05) on the product spectrum in such batch assays. In another set of experiments, three bioreactors were operated with continuous CO supply, in order to estimate the effect of running conditions on products and biomass formation. The bioreactors were operated under different conditions, *i.e.*, EXP1 (pH = 5.75, YE 1g/L), EXP2 (pH = 4.75, YE 1 g/L) and EXP3 (pH = 5.75, YE 0.2 g/L). When compared to EXP2 and EXP3, it was found that EXP1 yielded the maximum biomass accumulation (302.4 mg/L) and products concentrations, *i.e.*, acetic acid (2147.1 mg/L) and ethanol (352.6 mg/L). This can be attributed to the fact that the higher pH and higher YE concentration used in EXP1 stimulated cell growth and did, consequently, also enhance metabolite production. However, when ethanol is the desired end-product, as a biofuel, the lower pH used in EXP2 was more favourable for solventogenesis and yielded the highest ethanol/acetic acid ratio, reaching a value of 0.54.

## 1. Introduction

Carbon monoxide (CO) is emitted in large amounts in the form of industrial waste gases generated during the incomplete combustion of carbon-containing materials. It is also a major component of synthesis gas [1]. Some anaerobic bacteria have the ability to grow on CO as their sole carbon source and metabolize it to a variety of fuels and chemicals [2,3]. These unicarbonotrophs ferment CO into acetyl-CoA, via the acetyl-CoA pathway or Wood-Ljungdahl (WL) pathway, and later into metabolites such as acetic acid, ethanol, hydrogen, *n*-butanol or 2,3-butanediol. In the WL pathway, the net ATP gained by substrate level phosphorylation (SLP) is zero; hence, in order to make bacterial growth on CO possible, the WL pathway must be coupled to energy conservation [4,5]. However, the exact mechanisms involved in energy conservation remain still unclear. Very recently, metabolically engineered acetogens have been used to selectively produce metabolites from CO [6,7], although it is also possible to produce specific metabolites of interest from CO, in wild type bacteria, through manipulation of the medium composition and/or operating conditions in bioreactors [8,9]. Several acetogens are known to produce acetic acid, as major end metabolite, from CO, including *Moorella thermoacetica*, *Acetobacterium woodii*, *Eubacterium limosum* KIST 612, *Peptostreptococcus productus* U-1 and *Clostridium aceticum* [3]; whereas *Clostridium ljungdahlii*, *Clostridium autoethanogenum*, *Clostridium ragsdalei* and *Alkalibaculum bacchi* are ethanologenic acetogens, able to produce ethanol besides acetic acid [2,3]. Recently *Clostridium ljungdahlii*, *Clostridium autoethanogenum* and *Clostridium ragsdalei* were found to produce 2,3-butanediol and lactic acid as well [10]. 

In the present work, biological conversion of CO was studied, using *C. autoethanogenum*, in order to produce various metabolites. In most of the CO bioconversion studies to ethanol, co-production of large amounts of acetic acid was observed. Although ethanol is an interesting metabolite as a biofuel, products such as acetic acid have many industrial applications as well, as the key raw material for the manufacture of vinyl acetate monomer, acetic anhydride and acetate esters such as ethyl acetate, *n*-butyl acetate and isopropyl acetate [11]. Similarly, 2,3-butanediol is another possible by-product, with potential applications in manufacturing industries, such as in the production of food, pharmaceuticals, printing inks, perfumes, fumigants, synthetic rubbers, octane boosters, or plasticizers. Three stereoisomers of 2,3-butanediol exist, comprising the optically active dextro-[l-(+)-] and levo-[d-(−)-] forms and the optically inactive *meso*-form. It has been reported that *C. autoethanogenum* can produce 2,3-butanediol in the form of d(−)-2,3-butanediol (96%) and *meso*-2,3-butanediol (4%) [10]. This anaerobic biological route of production of chemicals such as ethanol, acetic acid and 2,3-butanediol from CO is an extremely attractive alternative compared to the traditional chemical route and other biorefinery processes [3]. 

Microorganisms require nitrogen for their structural integrity as well as for proteins, and optimization of their concentrations in culture media could improve the productivity of the process and reduce the medium’s cost. In some of our previous batch studies, it was found that the nature and the concentration of metabolites produced from CO depend on the composition of the culture medium as well as on other experimental conditions such as pH and pressure, among others [12]. Guo *et al*. observed that an optimized medium containing (g/L) NaCl 1.0, KH_2_PO_4_ 0.1, CaCl_2_ 0.02, yeast extract 0.15, MgSO_4_ 0.116 and NH_4_Cl 1.694, at pH = 4.74 could yield an ethanol concentration of around 0.25 g/L using *C. autoethanogenum* in microcosm studies [13]. Some previous study was done to evaluate the sensitivity of growth and product formation to nitrogen sources and their concentration in clostridia [14]. However, xylose was used as the carbon substrate in that study rather than CO. This prompted us to carry-out the present studies with CO, as the xylose fermentation by acetogens exhibits some differences and does also involve the glycolysis and oxidation of pyruvate to acetyl-CoA in addition to the WL pathway. Besides, the few previous studies aimed at estimating the effect of the medium’s composition on bacterial growth and production of metabolites in clostridia were generally done in batch assays, in bottles, with no pH regulation. In the present research, bioreactors operated at constant pH, with continuous CO supply, were used. This is a relevant aspect as both pH and the medium’s composition affect the metabolism and growth pattern. When both parameters are allowed to vary, it becomes difficult to conclude which one is actually affecting more. 

The purpose of this work was to investigate the effect of various sources of nitrogen on the bioconversion of CO to various metabolites, by *C. autoethanogenum,* in bottles as well as in continuous gas-fed bioreactors. In the present study, first, the influence of different sources of nitrogen (NH_4_Cl, yeast extract and trypticase soy broth) were compared for their effect on growth and product formation. In the research described in this paper, acetic acid is the major end-product. The adequate selection of the medium and culture conditions would allow ethanol to become the major, or even single, end metabolite. First, the experiments were carried out in 200 mL serum vials using a 2^3^ full factorial design. In the second part of the research the effect of individual sources of nitrogen on growth and metabolites production was studied. In the final part of the research, experiments were performed in laboratory-scale fermentors in continuous mode (continuous gas feed) applying results and conditions previously optimized in batch experiments.

## 2. Experimental Section 

### 2.1. Microorganism 

*Clostridium autoethanogenum* DSM 10061 was acquired from the Deutsche Sammlung von Mikroorganismen und Zellkulturen GmbH (Braunschweig, Germany), and was maintained on medium (pH = 6) with the following composition (per liter distilled water): NH_4_Cl, 0.9 g; NaCl, 0.9 g; MgCl_2_·6H_2_O, 0.4 g; KH_2_PO_4_, 0.75 g; K_2_HPO_4_, 1.5 g; FeCl_3_·6H_2_O, 0.0025 g; trypticase peptone, 2.0 g; yeast extract (YE), 1.0 g; cysteine-HCl, 0.75 g; 0.1% resazurin, 0.5 mL; with 0.5% xylose and SL-10 solution, 1.0 mL. The trace metal stock solution SL-10 contained (per liter): 7.7 M HCl, 10 mL; FeCl_2_·4H_2_O, 1.5 g; ZnCl_2_, 70 mg; MnCl_2_·4H_2_O, 100 mg; H_3_BO_3_, 6 mg; CoCl_2_·2H_2_O, 190 mg; CuCl_2_·2H_2_O, 2 mg; NiCl_2_·6H_2_O, 24 mg; and Na_2_MoO_4_·2H_2_O, 36 mg. For the experimental studies, xylose was omitted from the medium. 

### 2.2. Bioconversion Studies

#### 2.2.1. Bottle Batch Experiments

A two level three factor (2^3^) full factorial experimental design was used to study the combined effects of NH_4_Cl (0.2–2 g/L), trypticase (0.2–2 g/L) and YE concentrations (0.1–1 g/L), as sources of nitrogen, on products formation and culture stability during carbon monoxide bioconversion by *C. autoethanogenum*. The software package Minitab 16 (Minitab Inc. State College, PA, USA) was used to design the experiments and for data analysis in the form of analysis of variance (ANOVA). Table 1 shows the design matrix obtained in uncoded values with the MINITAB software and the observed values of the responses obtained for each experiment as well as the final pH. Factorial design is an important statistical tool that allows to conclude the factors that are most influential in the bioconversion process by carrying out a limited number of experiments. Thus, a total of 18 experimental runs, including the replicate experiments at the central points, were carried out. The individual and interaction effects of the different parameters were studied using the least square technique with the help of a specific software.

**Table 1:** 2^3^ Factorial design table of experiments and responses.

Run No	NH_4_Cl	Trypticase	YE	Ethanol (g/L)	Acetic Acid (g/L)	Biomass (mg/L)	Final pH
1	0.2	0.2	0.10	0.1733	1.806	152.29	3.88
2	2.0	0.2	0.10	0.3032	1.560	142.81	3.84
3	0.2	2.0	0.10	0.2290	1.855	222.7	4.03
4	2.0	2.0	0.10	0.1959	1.663	244.49	4.00
5	0.2	0.2	1.00	0.0883	2.146	302.90	3.91
6	2.0	0.2	1.00	0.1048	2.101	294.80	3.84
7	0.2	2.0	1.00	0.1061	2.339	335.62	3.93
8	2.0	2.0	1.00	0.1101	2.226	320.03	3.94

For batch experiments, 10% of actively growing seed culture, grown with CO as sole carbon source, was aseptically transferred into 200 mL serum vials containing 75 mL medium at pH = 6. The medium contained (per liter distilled water): NaCl, 0.9 g; MgCl_2_·6H_2_O, 0.4 g; KH_2_PO_4_, 0.75 g; K_2_HPO_4_, 1.5 g; FeCl_3_·6H_2_O, 0.0025 g; 0.1% resazurin, 0.5 mL; and SL-10 solution, 1.0 mL. NH_4_Cl, YE or trypticase were added in the same vials as per the experimental design (Table 1). In order to remove oxygen, the medium was boiled and flushed with N_2_. After cooling, 0.75 g cysteine-HCl, was added as reducing agent, and the pH was adjusted to 6 using aqueous solutions of either 2 M HCl or 2 M NaOH. The bottles were then sealed with Viton stoppers and capped with aluminum crimps before autoclaving for 20 min at 121 °C. The experimental set-up and the method used for media preparation are described elsewhere [15]. The bottles were maintained under anaerobic conditions. They were pressurized with 100% CO to reach a total headspace pressure of 1.2 bar and were agitated at 150 rpm on an orbital shaker, inside an incubation chamber at 30 °C. Headspace samples of 0.2 mL were used for CO measurements, and 1 mL liquid sample was periodically withdrawn from the vials, once every 24 h, in order to measure the optical density (OD_λ = 600 nm_), which is directly related to the biomass concentration. Afterwards, that same 1 mL sample was filtered using a 0.22 µm PTFE syringe-filter and was used to check the concentrations of soluble products. All the bioconversion experiments were conducted in duplicate, reaching statistically highly reproducible results. The response variables (Y) that were analyzed were the maximum products concentrations (g/L) as well as biomass concentrations (mg/L) obtained from the different experimental trials. 

Three separate experiments with either NH_4_Cl (1.1 g/L), YE (0.55 g/L) or trypticase (1.1 g/L), as sole source of nitrogen, were also performed in duplicate in order to understand the individual effect of each nitrogen source in promoting growth or product formation on CO. Another set of experiments, under the same conditions as above but without any CO, was also performed to check any product formation from YE and trypticase alone. The concentrations of nitrogen sources used in these sets of experiments are the center values of the respective factor ranges considered in the above full factorial design. Experiments and sample analysis were performed in the same way as mentioned above.

#### 2.2.2. Continuous Gas-Fed Bioreactor Experiments

Three bioreactor experiments were carried out in 2 L BIOFLO 110 bioreactors (New Brunswick Scientific, Edison, NJ, USA) using the following conditions: (1) pH = 5.75 and YE 1 g/L (called EXP1); (2) pH = 4.75 and YE 1 g/L (EXP2) and (3) pH = 5.75 and YE 0.2 g/L (EXP3). Those experiments were done with 1.2 L batch liquid medium and CO (100%) as the gaseous substrate, continuously fed at a rate of 15 mL/min using a mass flow controller (Aalborg GFC 17, Müllheim, Germany). The bioreactor with the medium was autoclaved and cysteine-HCl (0.75 g/L) was added after cooling, together with nitrogen feeding to ensure anaerobic conditions. The composition of the medium used in these bioreactor studies was the same as in the bottle experiments, with YE as the sole nitrogen source. The bioreactor was maintained at a constant temperature of 30 °C with a constant agitation speed of 250 rpm throughout the experiments. 10% of an actively growing culture, which was grown for 48 h with CO as sole carbon source, was used as the inoculum and was aseptically transferred to the bioreactor. The pH of the medium was automatically maintained at a constant value of either 5.75 or 4.75, through addition of either a 2 M NaOH solution or a 2 M HCl solution, fed by means of a peristaltic pump. Gas samples of 0.2 mL were taken from the inlet and outlet sampling ports of the bioreactor to monitor the CO and CO_2_ concentrations. Similarly, 2 mL liquid samples were periodically withdrawn from the reactor, once every 24 h, in order to measure the optical density (OD_λ = 600 nm_), allowing to estimate the biomass concentration. Afterwards the sample was filtered with a syringe using a 0.22 µm PTFE-filter before analyzing the concentrations of water-soluble products.

### 2.3. Analytical Equipment and Measurement Protocols

Gas-phase CO concentrations were measured using an HP 6890 gas chromatograph (GC, Agilent Technologies, Madrid, Spain) equipped with a thermal conductivity detector (TCD). The GC was fitted with a 15 m HP-PLOT Molecular Sieve 5A column (ID: 0.53 mm, film thickness: 50 μm). The oven temperature was initially kept constant at 50 °C, for 5 min, and then raised by 20 °C·min^−1^ for 2 min, to reach a final temperature of 90 °C. The temperature of the injection port and the detector were maintained constant at 150 °C. Helium was used as the carrier gas. Similarly, CO_2_ was analyzed on an HP 5890 gas chromatograph, equipped with a TCD. The injection, oven and detection temperatures were maintained at 90, 25 and 100 °C, respectively. For 2,3-butanediol identification, a Thermo Scientific ISQ™ single quadrupole GC-MS system (Thermo Fischer Scientific, Madrid, Spain ) was used and operated at 70 eV. It was equipped with a HP-5ms column (30 m × 0.25 mm × 0.25 µm film thickness). The water-soluble products in the culture broth, *i.e.*, acetic acid, ethanol and 2,3-butanediol, were analyzed using an HPLC (HP1100, Agilent Technologies, Madrid, Spain ) equipped with a 5 μm × 4 mm × 250 mm Hypersil ODS column and a UV detector at a wavelength of 284 nm. The mobile phase was a 0.1% ortho-phosphoric acid solution fed at a flow rate of 0.5 mL/min. The column temperature was set at 30 °C. Cell mass was estimated by measuring the absorbance of the sample, at a wavelength of 600 nm, using a UV–visible spectrophotometer (Hitachi, Model U-200, Pacisa & Giralt, Madrid, Spain). The measured absorbance was then compared to a previously generated calibration curve, to calculate the corresponding biomass concentration (mg/L). Besides, the redox potential was monitored continuously using an Ag/AgCl reference electrode connected to a transmitter (M300, Mettler Toledo, Inc., Bedford, MA, USA) and maintained inside the bioreactor.

## 3. Results and Discussion

### 3.1. Bottle Batch Experiments

**Figure 1:**
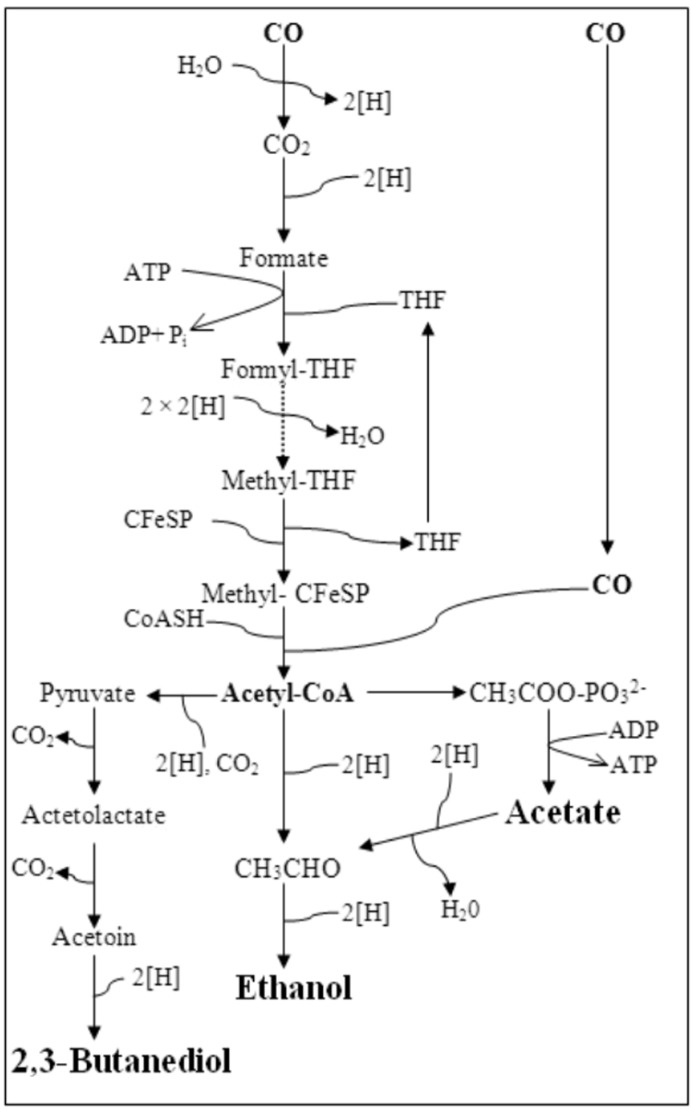
Wood-Ljungdahl pathway and metabolites formation from acetyl-CoA. Abbreviations: THF, tetrahydrofolate; CFeSP, corrinoid iron-sulphur protein.

In the bottle experiments, ethanol and acetic acid production started immediately, without any lag phase (Figure S1). It could be concluded that in these experiments the *Clostridium* strain follows the metabolic route that converts acetyl-CoA to acetaldehyde, followed by reduction to ethanol via a bifunctional acetaldehyde/ethanol dehydrogenase (Figure 1) [1]. Hence, in this CO fermentation, there were no differentiated acetogenic or ethanologenic phases. Maximum biomass (335.6 mg/L) and acetic acid concentrations (2.3 g/L) were produced in run No. 7 (Table 1) when the highest concentrations of YE and trypticase were used. The highest ethanol concentration (0.3 g/L) was obtained in run No. 2. Minor concentrations of by-product, *i.e.*, 2,3-butanediol, were also detected, reaching 0.017–0.101 g/L on the final day of the batch runs. The batch assays were stopped after about 10 days, when all the CO added initially was exhausted and no more biomass nor end-products were formed. 

#### 3.1.1. Main Effects Plot

**Figure 2:**
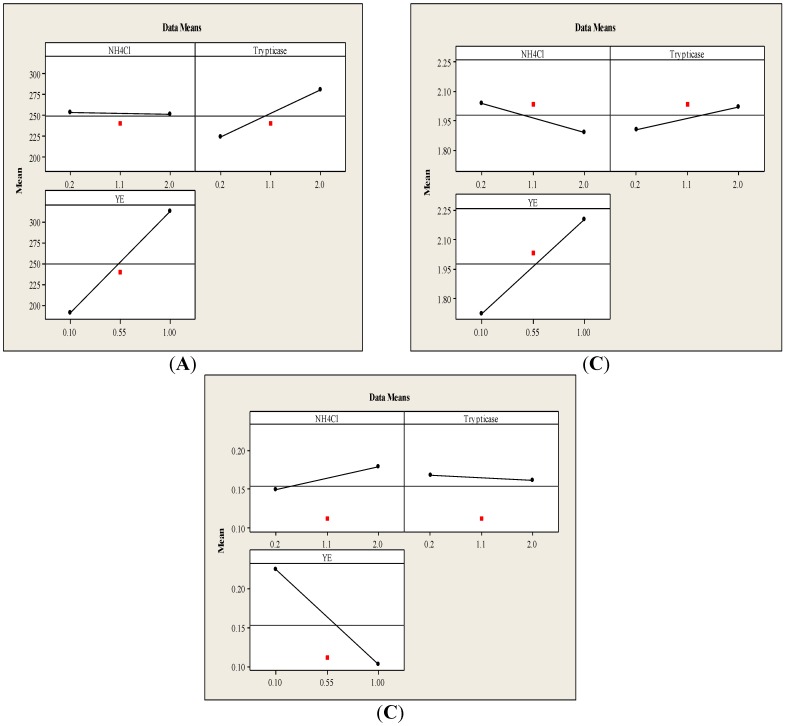
Main effects plot for (**a**) Biomass, (**b**) Acetic acid and (**c**) Ethanol.

The main effects plot for the experimental responses is shown in Figure 2. It represents the mean response values at each level of the design parameters. A main effect is considered present when the mean response changes across the level of the factor. From the main effects plot for biomass (Figure 2a), it is clearly observed that NH_4_Cl does not exert any significant effect on biomass. However, a slightly higher biomass concentration was observed whenever low NH_4_Cl concentrations were used in this study. This effect is in agreement with previously reported studies with *Clostridium aceticum* and *Rhodospirillum rubrum* using CO as the sole carbon substrate [16]. The presence of both NH_4_^+^ and acetate could presumably result in the formation of ammonium acetate which is inhibitory to some clostridia, already at low concentrations [17].

Based on the main effects plot (Figure 2a), cell growth of *C. autoethanogenum* was obviously affected by the initial YE and trypticase concentrations in the medium. The amount biomass increased with an increase of initial YE as well as trypticase concentrations within the range of concentrations studied in this work. This can be attributed to the nutritional value of YE and trypticase soy broth, as both contain various amino acids, vitamins and other growth-stimulating compounds. 

From the ANOVA analysis, it was observed that out of all the individual effects of each source of nitrogen, the effects due to the YE concentration was found statistically significant (*p* < 0.05) for ethanol and acetic acid production. For ethanol production (Figure 2c), the presence of YE showed the highest negative effect, whereas NH_4_Cl and trypticase exerted either a slightly positive or a slightly negative effect, respectively. The positive effect of NH_4_Cl on ethanol production was also reported by Guo *et al*., Plackett–Burman design was used in their studies, screening NH_4_Cl as one of the significant factors affecting ethanol production, along with MgSO_4_ and pH [13]. Enhanced growth in YE-limited media has been reported in previous studies. The presence of YE results in a richer medium, which is favorable for biomass growth. Biomass growth is usually related to acetate formation, while ethanol production is generally not a growth-related metabolite. Barik *et al.* suggested that a minimum level of approximately 0.01% YE would be essential for providing trace nutrients for cell growth. However, up to 300% improvement in the ethanol/acetate ratio was observed when YE was completely eliminated [18].

The negative effect of YE on ethanol production is expected to be due to vitamin B12, among others. YE contains vitamin B12, which plays an important role in acetogenic bacteria. Methyl transferase synthase (MeTr) in acetogens is a cobalamin-dependent enzyme and catalyzes the transfer of the methyl group of methyl-H4folate to the cobalt center of the corrinoid iron–sulfur protein (CFeSP). It is proposed that by reducing the H4folate cycle rate, NAD(P)H can build up inside the system with a subsequent increase in ethanol production [9]. In another study conducted with *Alkalibaculum bacchi* strain CP15, in a 7-L fermentor, a similar effect was observed; *i.e.*, a YE-free medium produced 13% more ethanol than a YE-containing medium. However, a decreased production of acetic acid and cell mass, reaching up to 40% and 15%, respectively, was observed in the YE-free medium [19].

#### 3.1.2. Interaction Effects Plot

**Figure 3:**
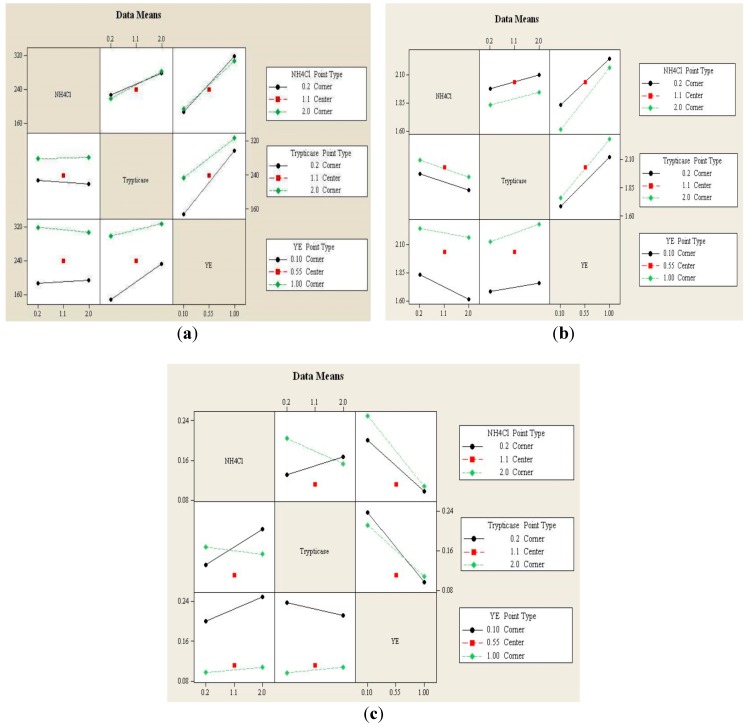
Interaction effects plots for (**a**) Biomass, (**b**) Acetic acid and (**c**) Ethanol.

The interaction effects plot for biomass, ethanol and acetic acid produced from CO is shown in Figure 3 and provides the mean response of all possible combinations from low to high level of each two factors. That is, the effect of each factor dependent upon the second factor. Non-parallel lines represent an interaction between those two factors (YE, trypticase, and/or NH_4_Cl). From the interaction plot for biomass and ethanol (Figure 3a,c), it can be observed that there is a strong interaction between each two factors. However, there is no remarkable interaction between the pairs of factors for acetic acid production (Figure 3b). 

The maximum concentrations of biomass and acetic acid achieved were above 290 mg/L and 2.1 g/L, respectively, in all the experiments in which a YE concentration of 1 g/L was used, irrespective of the concentrations of trypticase and NH_4_Cl in the medium (Figure 3a,b). The amounts ethanol produced reached their maximum values when YE was present at a low concentration of 0.1 g/L, irrespective of the concentrations of the other two factors (trypticase and NH_4_Cl) (Figure 3c). This shows the influence of the YE concentration on the spectrum of products obtained from CO conversion in *C. autoethanogenum*. Considering the interaction between NH_4_Cl and trypticase, a higher amount of biomass was found to be produced at a higher trypticase concentration of 2 g/L, at both levels of NH_4_Cl, which can be attributed to the complex nutrients present in trypticase (Figure 3a).

#### 3.1.3. Effect of Individual Sources of Nitrogen on Growth and Product Formation

Experiments were performed with either NH_4_Cl (1.1 g/L), trypticase (1.1 g/L) or YE (0.55 g/L), as the only source of nitrogen. It was observed that there is no growth nor product formation in bottles containing only NH_4_Cl. In the bottles with YE or trypticase, similar behaviours were observed, with growth reaching up to approximately 230 mg/L, and product concentrations of around 0.07 g/L for ethanol and 2 g/L for acetic acid. However, it is also worth recalling that the amount YE used in preparing the medium is half the amount of trypticase. From these observations, in the subsequent studies in continuous bioreactors, YE was chosen as the sole nitrogen source. Since YE and trypticase also contain other compounds besides nitrogen-containing ones, their potential use as substrates for the production of end-metabolites was checked. In that sense, in experiments performed without any CO, it was observed that the presence of YE or trypticase could be involved in approximately up to 10% of the total acetic acid produced in experiments containing CO as carbon source as well as YE and trypticase.

### 3.2. Continuous Gas-Fed Bioreactor Experiments

**Figure 4:**
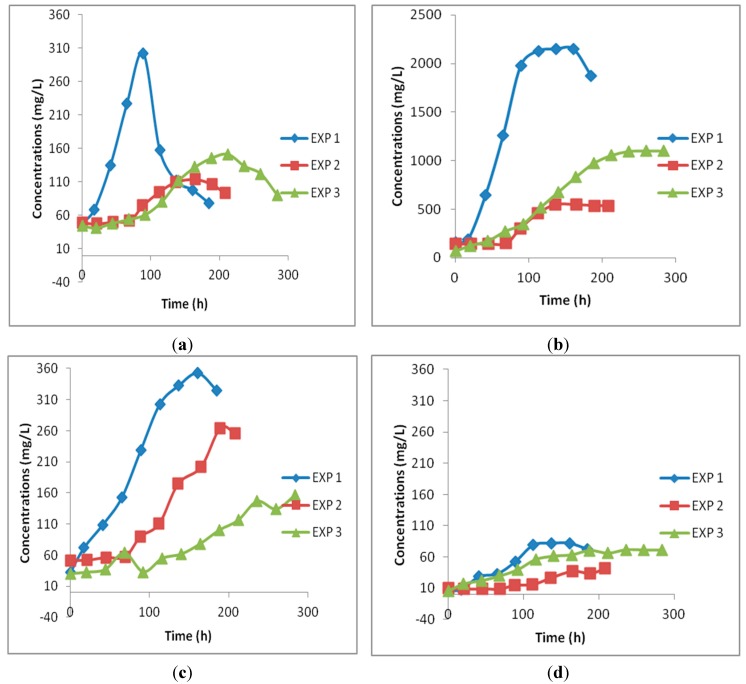
Cell mass (**a**) and products profiles, Acetic acid (**b**); Ethanol (**c**) and Butanediol (**d**) in three different experiments: EXP1 (pH = 5.75 and YE 1 g/L); EXP2 (pH = 4.75 and YE 1 g/L); EXP3 (pH = 5.75 and YE 0.2 g/L).

Bioreactor experiments with continuous gas-flow, *i.e.*, continuous CO supply, were performed for up to two weeks each. Cell growth and the production of different metabolites in three different sets of experiments are shown in Figure 4. The redox potential was constantly monitored for each experimental run. It is related to the electron transfer undergoing inside the cells and hence is very sensitive for even delicate changes in metabolism. Both EXP1 and EXP3 had an instrument reading oxidoreduction potential (ORP) value of −87 ± 10 mV, while it was −43 ± 5 mV for EXP2. The ORP values are directly dependant on the pH of the medium. A lower pH of the liquid phase will result in lower negative values of the redox potential. Oscillations of the redox potential values in the culture medium could be due to microbial growth and variations in the metabolic profile at each point of the experimental run and have also been reported by other researchers in other bioconversion studies [20,21]. Intracellular redox homeostasis is profoundly affected by the ups and downs of the extracellular redox potential which can significantly switch the fermentation type in acidogenic bacteria [22]. 

The biomass in EXP1 (Figure 4a) started growing after a shorter lag phase compared to EXP2 and EXP3, due to the favorable growth conditions (*i.e.*, optimal pH and nutritional value of YE) that prevail inside the bioreactor, attaining a biomass concentration of about 302.4 mg/L in less than 100 h of experimental run. The lag phase was approximately 70 h in both EXP2 and EXP3, reaching maximum biomass concentrations of 113.76 and 151.37 mg/L, respectively; that is 62% and 50% less than in EXP1. This confirms that the pH and YE concentration are important parameters and play a key role in achieving high cell mass concentrations. A drastic decrease in growth occurred after 89 h in EXP1. This could be linked to the accumulation of high amounts of acetic acid (~ 2 g/L) in the fermentation broth. Two enzymes are responsible for the conversion of acetyl CoA during the synthesis of acetate, *i.e.*, phosphotransacetylase (PTA) and acetate kinase (AK). During the acetate production stage, both enzymes are active and ATP is produced as a part of their reaction. However, it was reported that the activity of these enzymes decreases considerably with an increase in acetate concentration in the broth in fermentation with *C. acetobutylicum* [23]. In the latter study, the AK was biosynthesized inside the cell of *C. acetobutylicum*, with buildup of acetate concentrations of up to 3 g/L in the broth, resulting in a rapid decrease in the AK activity with the increase of the amount acetate [23]. However, a clear explanation for stoppage of growth and metabolite production in EXP2 and EXP3 after a certain period of time is yet somehow unclear. 

No separate acidogenic and solventogenic phase was observed for *C. autoethanogenum* during these bioreactor studies using the reported media compositions and fermentation conditions. The conversion of acetic acid to ethanol in the late phase of the study was also not observed, although we observed such type of conversion of acetate to ethanol under different operating conditions (manuscript in preparation). Acetic acid was the predominant metabolite formed during CO fermentation in each of the three experiments described here (Figure 4b). As mentioned above, changing the experimental conditions would allow a shift to ethanol accumulation rather than acetate. A maximum acetic acid concentration of 2.1 g/L was obtained after 137 h in EXP1, which is about 294% and 95% higher than the maximum amounts produced in EXP2 and EXP3, respectively. It is interesting to note that both experiments, EXP1 and EXP3,that were performed at high pH, produced more acetic acid than in studies at lower pH, irrespective of the YE concentrations used. A previous study using *C. ragsdalei* at two different pH values similarly reported a higher acetic acid production at high pH [24].

Although the maximum amount of ethanol was obtained in EXP1, the ratio ethanol/acetic acid was greater in EXP2 characterized by a low pH. Fermentation pH is one the most influential parameters that affects the metabolism of acetogenic bacteria. Lowering the pH appears to cause a shift in the product spectrum from acidogenic to solventogenic phase. The explanation lies in the permeation of the undissociated weak acid, acetic acid, through the cell membranes resulting in a lower internal pH due to the entry of H^+^ ions. Bacteria overcome this physiological stress by producing solvents [25].

As can be seen in Figure 4c, a higher maximum ethanol production was obtained in EXP1 than in EXP2, although the low external pH induced more solvent production. This could be due to the high biomass concentration achieved in EXP1. The fermentations produced 352.6 mg/L, 264.51 mg/L and 156.95 mg/L ethanol respectively in EXP1, EXP2 and EXP3. On the other hand, a maximum ethanol to acetic acid ratio was obtained for EXP2 with a value of 0.54. It can be seen that a low pH (EXP2) caused a lengthening of the lag phase and reduced the final biomass concentration, yet it significantly improved the ethanol/acetic acid ratio. Thus, nutrient limitation combined with a low fermentation pH improved such product ratio. Several studies reported that two-stage stirred tank bioreactors, with a different pH in each vessel could improve the ethanol to acetic acid ratio [26,27]. From this study it is observed that using a low initial pH and maintaining it constant could also improve the ethanol/acetic acid ratio, although there is a strong decrease in the overall productivity of metabolites. A major obstacle in CO fermentation, when focussing on ethanol production, is that lowering the pH reduces cell growth; thereby reducing the overall productivity of ethanol in the process. Minor amounts 2,3-butanediol were also produced in all three experiments (Figure 4d). The butanediol concentration increased to a maxium of 81.8, 41.8 and 71.6 mg/L in EXP1, EXP2 and EXP3, respectively. 

## 4. Conclusions 

From the experiments it is clearly observed that altering the medium’s composition as well as pH alters the product spectrum and biomass growth. From the batch studies, the YE concentration was found to have a significant effect on ethanol production. EXP1, at pH = 5.75 and a YE concentration of 1 g/L, produced a maximum amount of biomass (302.4 mg/L) and maximum concentrations of products, *i.e.*, acetic acid (2147.1 mg/L), ethanol (352.6 mg/L) and butanediol (81.8 mg/L), compared to the other two studies. A maximum ethanol to acetic acid ratio of 0.54 was obtained in EXP2 (pH = 4.75; YE 1 g/L). Though maintaining a low constant pH from the beginning improved the ethanol to acetic acid ratio, it drastically affects the overall productivity of the process as a result of a weaker biomass growth. 

## Supplementary Files

Supplementary File 1
