# Supplementary material for: Ethanol and Acetic Acid Production from Carbon Monoxide in a Clostridium Strain in Batch and Continuous Gas-Fed Bioreactors
Source: Int J Environ Res Public Health. 2015 Jan 20;12(1):1029–43. doi: 10.3390/ijerph120101029 (PMC4306909; doi:10.3390/ijerph120101029)
Supplement: Supplementary File 1 [file ijerph-12-01029-s001.pdf]

Supplementary Information

Ethanol and Acetic Acid Production from Carbon Monoxide in a *Clostridium* Strain in Batch and Continuous Gas-Fed Bioreactors

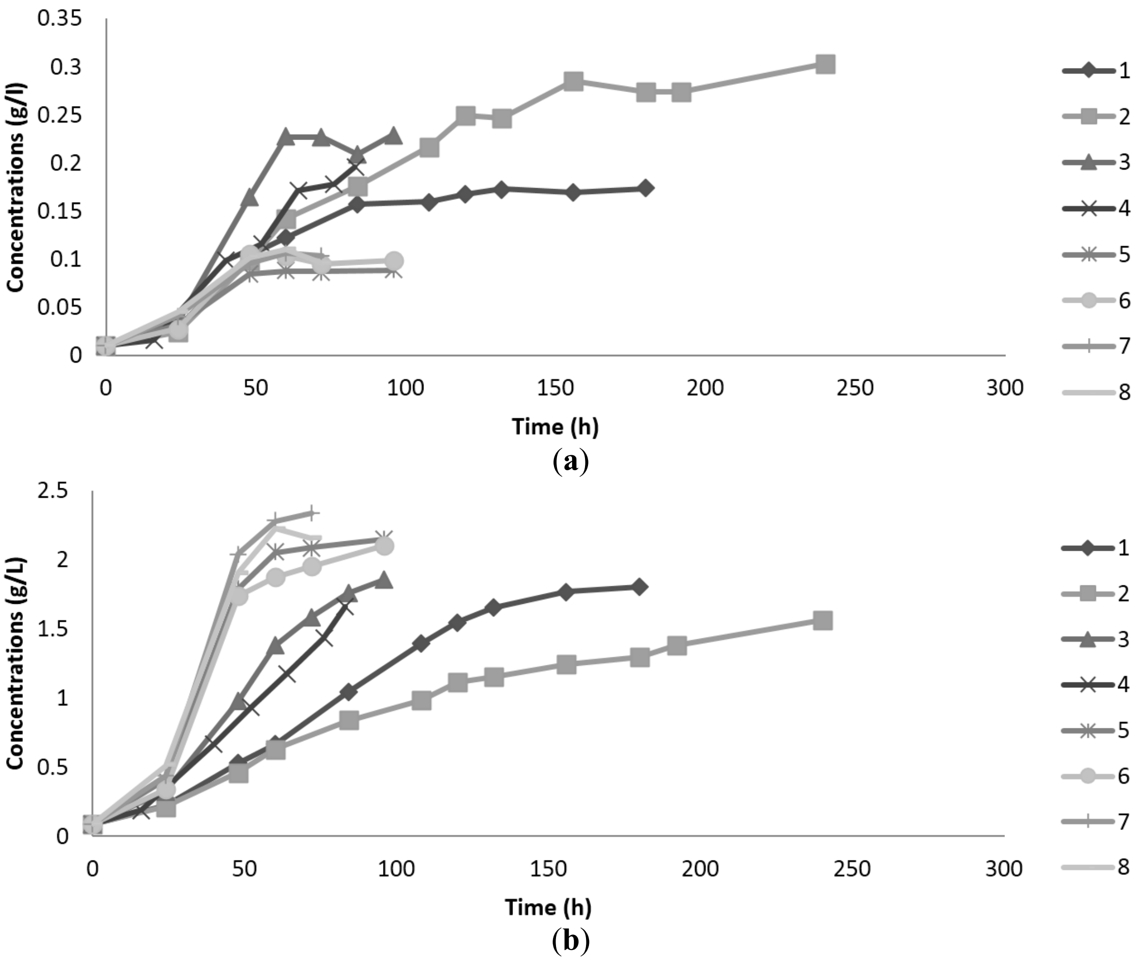

Figure S1. Products profile at eight different runs performed in bottle experiments: (**a**) ethanol profile and (**b**) acetic acid profile.

© 2015 by the authors; licensee MDPI, Basel, Switzerland. This article is an open access article distributed under the terms and conditions of the Creative Commons Attribution license (http://creativecommons.org/licenses/by/4.0/).
